# Pancreatic Cancer Risk Assessment Tools in Primary Care: A Mixed Methods Systematic Review

**DOI:** 10.1007/s12029-025-01229-5

**Published:** 2025-06-05

**Authors:** Hugh Claridge, Elizabeth A. Cooke, Spencer A. Thomas, Nan Greenwood, Agnieszka Lemanska

**Affiliations:** 1 School of Health Sciences, Faculty of Health and Medical Sciences, University of Surrey, Guildford, UK; 2 National Physical Laboratory, Teddington, UK; 3 Kingston University, Kingston, UK

**Keywords:** Primary care, Pancreatic cancer, Risk assessment tools

## Abstract

**Background:**

Pancreatic cancer is the twelfth most common cancer worldwide, but high mortality rates make it the sixth leading cause of cancer deaths. Diagnosis is frequently too late for curative intervention. Risk assessment tools incorporating diagnostic prediction models may assist early pancreatic cancer detection by primary care clinicians.

**Aim and Methods:**

This mixed methods systematic review aims to identify risk assessment tools which can be used for the detection of pancreatic cancer and have been investigated in primary care. It also seeks to synthesise the qualitative and quantitative evidence relating to the patient and clinician perspectives and experiences with these tools.

**Results:**

Ten studies were included with five risk assessment tools identified: ‘QCancer’, ‘eRATs’ (electronic risk assessment tools), ‘CaDet’, ‘Future Health Today’ and ‘C the Signs’. No tools were found for pancreatic cancer alone. Thematic synthesis of stakeholder perspectives resulted in three themes: impact on clinical decision-making, impact on patient consultations and implementation barriers and facilitators. Overall, experiences and impacts were positive, especially if used by less experienced clinicians.

**Conclusion:**

There is little evidence for the inclusion of many developed pancreatic cancer diagnostic prediction models in risk assessment tools in primary care, and limited research into stakeholder perceptions, especially patient perceptions. This review can inform future tool development, and further research should be undertaken assessing these tools’ clinical validity to encourage uptake in clinical practice.

**Study Registration:**

This study was registered prospectively as PROSPERO CRD42024488160.

**Supplementary Information:**

The online version contains supplementary material available at 10.1007/s12029-025-01229-5.

## Introduction

Pancreatic cancer is the twelfth most common cancer worldwide, with over 500,000 cases annually [1]. The prognosis for people diagnosed is often poor, with a median survival of 9 months and under 10% surviving beyond 5 years [2]. The non-specific nature of its early symptoms, including backache and fatigue [3], means many people eventually diagnosed have previously presented many times with symptoms to their general practitioner (GP) [4]. This contributes to delays in the diagnostic process leading to later stage diagnosis and reduced opportunities for curative treatment [5]. Therefore, any strategies that can facilitate early detection and diagnosis can be expected to provide benefits for patients and health services.

Risk assessment tools may achieve early detection of a number of health conditions, including pancreatic cancer [6, 7]. These tools can incorporate statistical diagnostic prediction models which calculate the risk of currently having the disease, triggering investigations [8, 9]. They do this by evaluating symptoms, risk factors and test results, the significance of which may otherwise be missed [10]. The intention is for these tools to enhance clinicians’ decision-making, rather than to replace or over-rule their judgement, enabling them to use the tools in conjunction with their experience and expertise [11]. A review of risk prediction models for pancreatic cancer found that 38 prognostic (risk of developing the disease in the future) and 52 diagnostic (risk of currently having the disease) prediction models have been developed [12]. However, it is unclear how many of these diagnostic models have seen clinical use in risk assessment tools.

Four systematic reviews investigating risk assessment tools in primary care for a range of health conditions, including cancers, have been published in the last 5 years [7, 13–15]. Together, these reviews provide useful general information relating to risk assessment tools in primary care, but their focus was not on tools for pancreatic cancer. Only one synthesised users’ experience of these tools, but only included patients and not clinicians’ perceptions [15]. This is important, because evidence from implementation science highlights the critical role of user experience in interacting with these tools which can help inform the development of future risk assessment tools for pancreatic cancer [16, 17]. In addition, the fast-paced development of pancreatic cancer risk prediction models [12] means an up-to-date review on risk assessment tools in primary care settings focussing on pancreatic cancer and patient and clinician perspectives is timely.

This mixed methods review aimed to identify risk assessment tools which can detect pancreatic cancer investigated in primary care worldwide and to synthesise the qualitative and quantitative evidence relating to the patient and clinician perspectives and experiences with these tools to inform future development of pancreatic cancer risk assessment tools.

## Methods

The review was conducted following good practice guidelines [18], using the Preferred Reporting Items for Systematic Reviews and Meta-Analyses (PRISMA) guidelines [19].

### Data Sources and Search Strategy

The search strategy was developed using the SPICE (Setting Population Intervention Comparison Evaluation [20]) approach (see Supplementary File 1 for SPICE criteria). Comprehensive searches were undertaken of the following electronic databases: Clarivate Analytics’ Web of Science (Web of Science Core Collection; BIOSIS Citation Index; BIOSIS Previews; Current Contents Connect; Data Citation Index; Derwent Innovations Index; KCI-Korean Journal Database; MEDLINE; Preprint Citation Index; SciELO Citation Index), Ovid Embase (excluding MEDLINE) and the Cochrane Library, all from inception to 11 October 2023, refreshed on 12 February 2024 and 17 January 2025.

The search strings used for Web of Science, Ovid and Cochrane are presented in Supplementary File 2. Searches consisted of a combination of terms related to cancer, primary care, risk assessment tools and terms relating to their use or reported experiences with these tools. Scoping identified that pancreatic cancer was not always mentioned in the title and the abstract; therefore, the final search terms were not restricted to pancreatic cancer. This required the retrieval and subsequent scrutiny of all identified studies which included a tool with a cancer risk assessment function, to ascertain if they incorporated pancreatic cancer.

To maximise opportunities for capturing relevant publications, three strategies were adopted. Google Scholar was used to perform forward and backward reference searching of studies fitting the inclusion criteria [21–23]. Backward reference searching of relevant reviews of cancer risk assessment tools identified in the database searches was also undertaken. Finally, as an enhanced search procedure, known pancreatic cancer risk prediction model development studies were also forward reference searched, to ascertain whether they have been reported as incorporated within risk assessment tools used in primary care.

### Inclusion and Exclusion Criteria

Broad inclusion criteria incorporating any peer-reviewed primary research were applied with no study design restrictions to reduce the chances of missing relevant studies and maximising the potential for generalisability [18]. No geographical, language, date or study design restrictions were applied. Studies were included if they reported the use of a risk assessment tool in a primary care setting by primary care professionals that could calculate the risk of the patient currently having undiagnosed pancreatic cancer. Tools only predicting future risk of developing cancer to guide prevention and behavioural advice were excluded.

Studies were excluded if they reported the use of risk assessment tools outside primary care and without involvement of primary care professionals. Literature reviews (including systematic reviews), protocols, PhD theses, opinion papers and grey literature were also excluded.

### Study Selection

Titles and abstracts retrieved from the electronic searching were uploaded to Rayyan.ai [24] with duplicate checking undertaken manually. To ensure consistency in inclusion and exclusion criteria interpretation, dual pilot screening was performed by two reviewers (HC, NG) for the first 500 results [25]. Titles and abstracts were then screened for retrieval and potential inclusion (lead author HC 100%, second reviewer NG 31%), with any disagreements resolved through discussion. The full texts selected for retrieval and potential inclusion were then screened independently by the two reviewers, with disagreements resolved through discussion. Additional studies identified by forward and backward citation searching were subjected to the same inclusion and exclusion criteria and study selection process.

### Data Extraction, Analysis and Synthesis

Data were extracted using a form specifically developed for this systematic review. The lead author (HC) extracted the data, and the extracted data were checked by a second reviewer (NG) against the source material. Data included study design, methodology, risk assessment tool, theoretical approach and analysis and themes developed relating to clinician and patient perspectives on their experiences with these tools. See data extraction Tables 1, 2, 3 and 4.

**Table 1:** Details of included studies

**Publication**	**Geographical location**	**Data collection dates**	**Risk assessment tool**	**Study aim(s)**	**Design and methods**	**Primary care setting and participant numbers with details**	**Sampling methods**	QualSyst rating*
Akanuwe et al*.* [26]	Lincolnshire, UK	2016	QCancer	To ‘explore the perspectives of service users and primary care practitioners on communicating cancer risk information to patients, when using QCancer….’ P510	Qualitative Semi-structured 1:1 interviews and focus groups	17 GP practices 17 GPs and practice nurses 19 ‘Service Users’	Convenience	15/20
Akanuwe et al*.* [27]	Lincolnshire, UK	2014–2015	QCancer	To ‘…explore service users’ and primary care practitioners’ perspectives on the barriers and facilitators to implementing a cancer risk assessment tool (RAT), QCancer, in general practice consultations.’ P1	Qualitative Semi-structured 1:1 interviews and focus groups	17 GP practices 17 GPs and practice nurses 19 ‘Service Users’	Convenience	15/20
Black et al*.* [28]	North East London, UK	2020–2022	C the Signs	To evaluate ‘…the use of E-SN software in primary care, examining its benefits and barriers, safety implications, and overall impact on individual and practice usage.’ P1	Qualitative Semi-structured 1:1 and 2:1 interviews	Number of GP practices not reported 15 GPs 5 administrative staff 1 practice nurse	Convenience	15/20
Chiang et al*.* [29]	Victoria, Australia	Not reported	QCancer	‘…. to explore the use of a cancer risk tool, which implements the QCancer model, in consultations and its potential impact on clinical decision making.’ S77	Qualitative Semi-structured 2:1 interviews following simulated patient consultations	Number of GP practices not reported 15 GPs	Purposive	18/20
Chima et al*.* [30]	Melbourne, Australia	2021	Future Health Today	‘…to explore the usefulness and feasibility of a novel quality improvement (QI) tool in which algorithms flag abnormal test results that may be indicative of undiagnosed cancer.’ P1	Qualitative Semi-structured 1:1 interviews and focus groups	GP clinics Interview participants: 9 GPs 4 practice nurses 1 practice manager Professional focus group participants: 6 GPs 2 practice nurses Consumer focus group participants: 10 consumers	Purposive	13/20
Chima et al. [31]	Victoria and Tasmania, Australia	2021–2022	Future Health Today	‘…to determine whether the FHT cancer module, coupled with education, training, and practice support, increased the proportion of patients receiving guideline-based care at 12-months post-randomisation, compared to an active control.’ P5	Quantitative Proportion of patients receiving guideline-based care at 12-months post-randomisation, compared to an active control	40 GP practices 7555 patients	Pragmatic cluster random sampling	26/26
Chima et al. [32]	Victoria and Tasmania, Australia	2022	Future Health Today	‘Explore the acceptability and usability of the FHT cancer module, which flags patients with abnormal test results that may be indicative of undiagnosed cancer.’ P1	Qualitative Semi-structured 1:1 interviews	12 GP practices 10 GPs 7 practice nurses	Convenience	17/20
Heller et al. [10]	Tel Aviv, Israel	Not reported	CaDet	‘…to compare the performance of the CaDet computer program in the identification of these clinical profiles to that of experienced clinicians.’ P353	Quantitative Comparison of clinicians’ opinions and CaDet’s outputs on summary of patient reported information	1 special primary care service within a medical centre 5 clinicians 160 patients	Convenience	14/18
Price et al. [33]	UK	2017	QCancer and eRATs	‘To quantify the availability and use of cancer decision-support tools (QCancer® and risk assessment tools) and to explore the association between tool availability and 2-week-wait (2WW) referrals for suspected cancer.’ P437	Quantitative Postal survey	227 GP practices 476 GPs and registrars	Random probability sample	21/22
Spear and Knight-Davidson [34]	East of England, UK	2022–2023	C the Signs	‘…to understand the perceived benefits and challenges of using C the Signs for safety netting in primary care, from the perspective of users of the system.’ P3	Qualitative Semi-structured 1:1 interviews and qualitative questionnaire	4 GP practices 10 GPs 1 practice nurses 1 paramedic 3 administrators	Convenience sample from purposively sampled GP practices	16/20

*QualSyst rating denominators vary depending on the relevance of the QualSyst criteria

**Table 2:** Risk assessment tool details

**Tool name**	**Underlying model(s)**	**Cancers included**	**Purpose**	**Used by study**	**Format of tool used in study**
CaDet	Fuchs et al. [35]	Multiple, including pancreatic	Cancer risk assessment	Heller et al. [10]	Patient questionnaire responses fed into CaDet programme
C the Signs	NICE guidelines and unnamed artificial intelligence model	Multiple, including pancreatic	Electronic safety netting, with cancer risk assessment	Black et al*.* [28], Spear and Knight-Davidson [34]	Software integrated within an electronic safety netting programme integrated within EMIS or SystmOne EHR management systems
Future Health Today	Guideline-based and unnamed model integrating recent research	Multiple, including gastrointestinal which includes the pancreas	Point of care prompts with guideline-based recommendations, and audit and recall tool to identify patients who may be at risk of an undiagnosed cancer using the results of abnormal tests and patient information	Chima et al*.* [30], Chima et al*.* [31], Chima et al*.* [32]	Electronic, integrated within individual electronic patient records, and a web-based portal with practice-wide audit and recall tools
eRATs	Stapley et al. [36]	Multiple, including pancreatic	Cancer risk assessment	Price et al. [33]	Integrated within GP software systems
QCancer	Hippisley-Cox and Coupland [37, 38]	Multiple, including pancreatic	Cancer risk assessment	Akanuwe et al. [26]	Vignette, demonstrated on web-based version where access available
				Akanuwe et al*.* [27]	Vignette, demonstrated on web-based version where access available
				Chiang et al*.* [29]	Web-based version, interface designed as a browser page
				Price et al*.* [33]	Integrated within GP software systems

**Table 3:** Theoretical approach, data collection and analysis of the studies included in the thematic synthesis

**Authors**	**Theoretical approach to the study**	**Data collection tool**	**Clinical vs. simulated tool experience**	**Data analysis**
Akanuwe et al*.* [26]	Risk analysis framework (RAF)	Interview guide informed by RAF	Simulated: participants used vignettes for clinical cases and tool use for the study	Framework analysis informed by RAF with further inductive coding
Akanuwe et al*.* [27]	Consolidated Framework for Implementation Research (CFIR)	Interview guide informed by CFIR	Simulated: participants used vignettes for clinical cases and tool use for the study	Framework analysis informed by CFIR with further inductive coding
Black et al*.* [28]	Not reported	Interview guide	Clinical: participants had previous clinical experience of the tool	Framework analysis informed by rapid appraisal procedure (RAP) and research questions
Chiang et al*.* [29]	Normalisation process theory (NPT)	Interview guide informed by NPT	Combination: participants used the tool with actors as patients	Framework analysis informed by NPT with further inductive coding
Chima et al*.* [30]	Clinical Performance Feedback Intervention Theory (CP-FIT)	No information on, e.g. interview or focus group topic guides reported	Combination: some participants had clinical experience whilst others had an online demonstration	Combination of inductive thematic analysis and a deductive approach using CP-FIT
Chima et al*.* [32]	Clinical Performance Feedback Intervention Theory (CP-FIT)	Interview guide informed by CP-FIT	Clinical: participants had previous clinical experience of the tool	Deductive content analysis with themes mapped onto CP-FIT
Price et al. [33]	Not reported	Survey designed using best practice guidelines and topic experts	Clinical: participants had previous clinical experience of the tool	Combination of descriptive and inferential statistics
Spear and Knight-Davidson [34]	Not reported	Interview guide and qualitative questionnaire developed from interview guide	Clinical: participants had previous clinical experience of the tool	Combination of deductive and inductive thematic analysis, using a framework specifically developed for the study

**Table 4:** Thematic synthesis

**Analytical theme**	Descriptive code*	**Study**
Impact on clinical decision making: positive	- Reduce over-referral	Akanuwe *et al. *[27]
	- Aids referral decisions	Chiang *et al. *[29] Spear and Knight-Davidson [34]
	- Prompting referrals that would otherwise not have been made (14 %)	Price *et al*. [33]
	- Prompting additional relevant investigations	Spear and Knight-Davidson [34]
	- Encourages review of clinical history	Chima *et al.* [32]
	- More efficient test result presentation	Chima *et al.* [32]
	- Confidence & reassurance in interpreting test results	Black *et al. *[28]
	- Useful for less experienced practitioners	Black *et al. *[28]
		Chiang *et al. *[29]
	- Helps with unusual & non-specific symptom presentation	Black *et al. *[28]
		Chiang *et al. *[29]
	- Assessing cancer risk in patients with: Non-specific symptoms (35 %); multiple symptoms (36 %)	Price *et al*. [33]
	- Reminder of existing guidelines	Black *et al. *[28]
		Chima *et al. *[30]
	- Helps prioritise clinical decisions	Chiang *et al. *[29]
	- Increasing the certainty of clinical decision making (12 %)	Price *et al*. [33]
	- Ensures timely action	Chima *et al. *[30]
		Chima *et al.* [32]
		Chiang *et al. *[29]
	- Increasing the awareness of cancer symptoms (12 %)	Price *et al*. [33]
	- Increasing the awareness of cancer as a possible diagnosis (22 %)	Price *et al*. [33]
	- Increases clinical knowledge & skills	Chima *et al.* [32]
Impact on clinical decision making: negative	- Over-referrals thus over-burdening services	Akanuwe *et al. *[27]
	- Sometimes prompts actions already taken	Chima *et al.* [32]
Impact on clinical decision making: recommendations	- Ensure it permits use of clinicians’ own judgement	Chima *et al. *[30]
		Chima *et al.* [32]
	- Present diagnostic guidance first, with secondary access to numerical risk if needed	Chiang *et al. *[29]
	- Make recommendations more concise	Chima *et al. *[30]
Impact on consultations: positive	- Provides simple, personalised risk information aiding understanding [patients & clinicians]	Akanuwe *et al. *[26]
		Akanuwe *et al. *[27]
	- Improves speed of assessment process [patients & clinicians]	Akanuwe *et al. *[27]
	- Enables shared decision making	Black *et al. *[28]
	- Numerical output reassures patients at low risk & aids clinician discussion with those at high risk	Chiang *et al. *[29]
	- Discussing cancer risk with a patient (29 %)	Price *et al*. [33]
	- Reassuring anxious patients (17 %)	Price *et al*. [33]
	- Discussing investigation with symptomatic patients (5 %)	Price *et al*. [33]
Impact on consultations: negative	- Increased consultation time needed to complete assessment and/or discuss implications with patients [patients & clinicians]	Akanuwe *et al. *[26]
		Akanuwe *et al. *[27]
		Chiang *et al. *[29]
		Chima *et al. *[30]
		Chima *et al.* [32]
		Spear and Knight-Davidson [34]
	- Challenges of communicating results	Chiang *et al. *[29]
	- Relative rarity of cancer thus causing unnecessary anxiety for patients	Chiang *et al. *[29]
		Akanuwe *et al. *[27]
Impact on consultations: recommendations	- Involve patient when using the tool to maintain trust [patients & clinicians]	Akanuwe *et al. *[26]
	- Reconsider risk output layout [patients & clinicians]	Akanuwe *et al. *[26]
	- Provide clearer explanations of how to understand the risk output [patients]	Akanuwe *et al. *[26]
	- Provide patients with the tool in the waiting room prior to consultation	Chiang *et al. *[29]
	- Traffic light colour coding as opposed to numerical risk	Chiang *et al. *[29]
	- Provide relevant patient resources to facilitate clinician-patient communication [patients & clinicians]	Chima *et al. *[30]
Implementation barriers	- Practitioner concerns about the validity & utility	Akanuwe *et al. *[27]
		Black *et al. *[28]
		Chiang *et al. *[29]
		Chima *et al. *[30]
	- Limited perceived advantage over prior knowledge & experience	Black *et al. *[28]
		Chiang *et al. *[29]
		Spear and Knight-Davidson [34]
	- Established process habits are sometimes preferred	Chima *et al.* [32]
	- Training needed for use	Akanuwe *et al. *[27]
		Chiang *et al. *[29]
		Spear and Knight-Davidson [34]
	- Some system features present a hinderance	Spear and Knight-Davidson [34]
	- Perceived lack of relevance to certain clinical roles	Chima *et al.* [32]
	- Audit element took too much time	Chima *et al. *[30]
		Chima *et al.* [32]
	- Competing priorities & insufficient resources to act on tool recommendations	Chima *et al.* [32]
	- None of the suggested functions considered useful (29 %)	Price *et al*. [33]
Implementation facilitators	- Point of care aspect subtle, nondisruptive & time efficient	Chima *et al. *[30]
	- Simple & efficient to use before & during consultations	Chima *et al.* [32]
	- Having confidence in the recommendations made	Chima *et al.* [32]
Implementation recommendations	- Link tool to clinical software	Akanuwe *et al. *[26]
		Akanuwe *et al. *[27]
		Chiang *et al. *[29]
	- Delegate specific functions within the tool to specific clinical roles to be time efficient	Chima *et al. *[30]

*Reported by clinicians only, unless otherwise marked with [patients] or [patients & clinicians].

A pragmatic approach with thematic synthesis was adopted, which included a mixture of deductive and inductive approaches [39, 40]. This method was selected because the included studies were methodologically heterogeneous with different reporting styles, and thematic synthesis offers a clear and transparent means for synthesising data from both qualitative and quantitative research [41]. An objective of this review was to synthesise the patient and clinician perspectives and experiences with these tools, and this guided the overall analysis. The analysis process was iterative and involved familiarisation with the selected studies through repeated reading whilst developing descriptive codes. The initial descriptive codes developed were then applied to the included studies and were refined with new codes developed as the analysis progressed. These descriptive codes were then developed into analytical themes, focussed on patient and clinician experiences and perspectives on the use of these tools [40]. These themes are presented in Table 4 and Supplementary File 5 Fig. 2. Selected quotes are included in the text to support these themes.

### Quality Assessment

The QualSyst tool [42] was used to critically appraise the included studies (see Supplementary File 3 for the QualSyst checklists). QualSyst was chosen as it provides standard criteria for evaluating primary research papers from a variety of fields and methodologies. Quality appraisal was conducted independently by the lead author (HC) and second reviewer (NG). The minor discrepancies were resolved by discussion. Studies were not excluded or weighted based on the quality scores, with the process of quality assessment used for enhancing study interrogation [39, 43].

## Results

Electronic searches identified a total of 9085 records before duplicate removal (7976 on 12 October 2023, additional 370 on 12 February 2024 and 739 on 17 January 2025) (see Supplementary File 4 Fig. 1 PRISMA diagram). After duplicate removal, the titles and abstracts of the remaining 8081 were screened. Of these, 30 were sought for full-text retrieval, with 29 successfully retrieved. Of these, nine studies fitted the inclusion criteria [26–34]. Forward and backward citation searching of the nine included studies and backward citation searching of relevant systematic reviews identified 37 potentially relevant records. After reviewing the full texts of these, one additional study met the inclusion criteria [10], giving a total of ten included studies.

When considering the findings, it is important to note that some publications report results from the same studies. Chima and colleagues had three relevant publications [30–32], with two reporting on different aspects of the same trial [31, 32]. Akanuwe et al. [26, 27] reported findings from the same participants but analysed by the authors using different theoretical approaches. These studies have therefore been treated separately.

Eight of the ten studies were published in the last 6 years (2019–2024). Three used quantitative methods [10, 31, 33], with seven adopting a qualitative approach [26–30, 32, 34] (Table 1).

Study participants’ experience with these tools varied. In some, the participants had prior clinical experience with the tools [28, 31–34], and although some participants in Chima et al. [30] also did, others received online demonstrations. In Chiang et al. [29], clinicians had access to a modified version of the tool and applied it in consultations with actors using vignettes. In Akanuwe et al. [26, 27], participants were given vignettes demonstrating how the tool could be used. Therefore, some findings relate more to anticipated rather than actual experience with the tools.

All studies were set in general practice in developed nations (the UK, Israel and Australia). Akanuwe et al*.* [26, 27] and Chima et al*.* [30] included both patient and clinician perspectives, whilst Chiang et al. [29], Price et al*.* [33], Black et al*.* [28], Chima et al. [32] and Spear and Knight-Davidson [34] covered only clinician perspectives. Five of the seven qualitative studies used theoretical frameworks to guide their study and to analyse their results [26, 27, 29, 30, 32]. Tables 1, 2 and 3 give further details of these studies.

### Risk Assessment Tools Identified

In the ten studies, five risk assessment tools were identified: ‘QCancer’ was reported most frequently [26, 27, 29, 33], followed by ‘Future Health Today’ (FHT) [30–32], with ‘eRATs’ (electronic risk assessment tools) [33], ‘CaDet’ (Cancer Detection) [10] and ‘C the Signs’ [28] each used in one study. No tools were found for pancreatic cancer alone. Table 2 provides more information on these risk assessment tools.

All the qualitative studies investigated participant perspectives of the use of these risk assessment tools in primary care, but their focus varied. The majority of these investigated the primary care professionals’ perspectives on the usability, facilitators and barriers to the use of these tools [27–30, 32, 34] except Akanuwe et al*.* [26] whose focus was specifically on communicating cancer risk. Focussing on the quantitative studies, Price et al. [33], as part of a larger study, surveyed primary care professionals’ perceptions of the usefulness of QCancer and eRATs. Chima et al*.* [31] examined the use of Future Health Today’s impact on follow-up rates. Heller et al. [10] examined the concordance between the cancer risk assessment of the risk assessment tool CaDet and primary care clinicians’ opinions on patients’ reported symptoms.

The qualitative studies performed well in their quality assessment with most scoring at least 15 out of 20 [26–29, 32, 34] (Table 1). The most common concerns with the qualitative studies related to sampling approaches and not reporting researcher reflexivity. Convenience sampling, which is generally regarded as more prone to selection bias than purposive sampling [44, 45], was deployed in five [26–28, 32, 34]. Two reported using purposive sampling, but only one [29] specifically described the characteristics included in this sampling approach, with Chima et al. [30] not detailing or explaining the selected characteristics. All qualitative studies omitted exploration of researcher reflexivity—the role of the interviewer in relation to the interviewee and any impact this could have on the data. Overall, Chima et al*.* [31], a quantitative study, scored highest of all included publications with full marks, closely followed by Price et al*.* [33] with only one assessment item docked for failing to achieve their target sample size.

Two publications were not included in the thematic synthesis [10, 31]. This is because the aim of this review was to identify tools reported as used in primary care settings and to synthesise the patient and clinician perspectives. Heller et al. [10] described the use of CaDet in this setting and examined the concordance between the cancer risk assessment of CaDet with primary care clinicians’ opinions on patients’ reported symptoms. Chima et al. [31] described the use of Future Health Today in this setting and examined the impact on follow-up rates. Neither reported on patient or primary care professionals’ experiences of these tools.

### Thematic Synthesis

Overall, study participants’ perceptions and experiences varied. Exploration of the eight studies included in the thematic synthesis led to the development of three themes relating to patient and clinician experiences and perceptions of the use of these risk assessment tools: impact on clinical decision-making, impact on patient consultations and implementation barriers and facilitators (Table 1). Price et al.’s [33] quantitative survey focussed only on the positive features of these tools, meaning that no negative features were highlighted. However, importantly, 29% of the GP respondents with access to electronic risk assessment tools (QCancer or eRATs) considered none of the functions highlighted by the survey to be useful.

Selected quotes from the included studies are provided to illustrate aspects of the themes, with some of these shortened for succinctness.

### Analytical Themes

#### Impact on Clinical Decision-Making

The most prominent theme related to these tools’ potential and mostly positive impact on clinical decision-making. Clinicians identified these tools as having a number of perceived advantages and very few disadvantages. The advantages include providing support assessing patients with unusual or multiple, non-specific symptoms [28, 29, 33], practitioner reassurance through increased confidence in test result interpretation [28], enhancing general clinical decisions such as increasing awareness of cancer as a possible diagnosis and prompting referrals or tests that may have otherwise not have been made [32–34]. In addition, they were seen to remind clinicians of existing guidelines and thus potentially increase concordance with them [28, 30].

*“So, I would be using these tools for re-educating me and ensuring I’m doing best practice because I’m getting older now and things change.”* [GP] [30]

These tools could also aid referral decisions [29, 34], reduce over-referrals [27] and encourage timely action [29, 30, 32].

*“Normally I’d get a few investigations, get the results back and then based on that say do we need to do something, or I refer this on based on that. But I guess if I have a calculator saying it’s higher risk, it might prompt me to make a referral to a specialist a bit earlier.”* [GP] [29]

Only one negative impact on clinical decision-making was highlighted with some clinical and patient participants in Akanuwe et al. [27] suggesting these tools may lead to over-referrals and over-burdening the UK’s National Health Service.

*“It could lead to over-referral as some people may have a certain risk but will not have cancer after they have been referred and tested.”* [Patient] [27].

Some participants also emphasised that care needed to be taken when developing the tools. Chima et al.’s [30] clinical participants recommended caution with the language used in the tool, ensuring the guidance was concise, contained the necessary resources and information for action, but not overly prescriptive so as to permit incorporation of clinicians’ judgement. Interestingly, in their more recent study, clinicians using the revised version of the same tool highlighted how succinct and usable the presented information was, facilitating clinical autonomy [32].

Overall, the advantages to clinical decision-making appeared to be more pertinent to less experienced primary care professionals [28, 29].

*“I guess as a trainee it is useful for me at times. I can think back to a sarcoma where I wasn’t really sure and the symptom decision tool was helpful. But most of the time it is more of a validation that what you thought was correct …”* [GP trainee] [28].

#### Impact on Patient Consultations

Another theme was the impact of these tools on patient-clinician consultations. In general, the impact here was also perceived to be more positive than negative. Advantages included expediting the consultation process [27, 30] and providing opportunities for shared decision-making and open discussions of patient cancer risk [28, 33]. In order for this to work well, it was recommended by some patients and clinicians that patients be involved throughout when using the tool [26].

*“I will like to be involved and I will like to see them using the tool … I will expect them to then explain to me what the results mean in terms of my risk.”* [Patient] [26]

This also highlighted the need to ensure the presentation of the risk output can be tailored to both patients’ and clinicians’ preferences [26], such as with both traffic light and numerical risk presentations [29].

*“… and then maybe not even using the word risk, you know, that’s where the colour coding might come in. You know if you saw something in green, I think as patient, you probably think, “That’s pretty—that’s not too bad”. And maybe you know, if it’s not green, you know, yellow might just be warning …”* [GP] [29]

The personalised nature of these assessments was also seen as beneficial [26, 27].

*“… you’re presenting them with their own risk not a general risk, it’s personal to them and it will just make the consultation more patient focused, and I think it will make patients feel more involved in the consultation and just feel more cared for.”* [Patient] [27].

A numerical risk output was thought to help reassure low risk patients and to encourage reluctant patients with high scores to consider further testing [29].

*“… they’re coming back in just for reassurance and I’ll say okay, you’ve done all those things, let’s just put your numbers in and let’s make sense of this, and then we’ll see what tests we have to do.”* [GP] [29]

Despite these positives, some patients and clinicians suggested that using the tools required additional time to both use the tools and to discuss the results and their implications [26, 27, 29, 30, 32, 34].

*“You wouldn’t want to feel that you’ve been rushed, you would want them to take time to talk with you, and if they try to cut this conversation short you would think that they didn’t care…”* [Patient] [26]

Compounding this, being unable to claim costs for actioning some of the recommendations was also seen as restricting use [32].

*“We’ve got nurses that can do it but that doesn’t have any Medicare item numbers and no funding, in a new private general practice it brings you no money, so we can’t really keep doing that.”* [GP] [32]

Given the relative rarity of cancer compared to other conditions and the fear the term cancer can bring, some participants noted the potential for unnecessary anxiety when patients are informed of the risk outputs [27, 29].

*“I then think because cancer, I think, is far more emotive than a risk of having a heart … attack … and it doesn’t worry them because everyone survives heart attacks these days … but cancer’s still got a really bad rep even though we treat it so well.”* [GP] [29].

Highlighting the diversity of opinion, some clinicians in Price et al*.*’s [33] survey thought using the tools reassured anxious patients. Furthermore, some patients in Akanuwe et al*.* [26] said they would prefer to be told about the use of the tool, even if it potentially increased their anxiety.

*“… A better way of using it [the tool] is that you should be told that they are using it. Even though that will still make you worry, I think that if it were something to worry it will be slightly different anyway.”* [Patient] [26]

#### Implementation Barriers and Facilitators

The included studies highlighted a range of perceived barriers to the use of cancer risk assessment tools in primary care. This theme prominently featured professionals’ scepticism. This included lack of confidence in the tools’ risk outputs and the dearth of evidence relating to the benefits of their use compared to standard clinical practice, and thus their added value to consultations [27–30].

*“We have to make sure that it is better than what we are already doing.”* [GP] [27].

Professionals’ lack of confidence in using the tools may have been demonstrated by the desire for training in the tools’ application and interpretation [27, 29, 30, 34].

*“[The tool] has massively more potential than what we’re all using it for. And if we could have protected time to really navigate it and then have a, you know, a review on it, then I think that might be a way of getting, you know, colleagues to use it more. […] I don’t feel we use it optimally, but that’s a time capacity issue.” *[GP] [34]

Perhaps supporting this, very few respondents to Price et al*.’*s [33] survey had received training in their use.

Experience appeared to play a part in the willingness to incorporate the tools into practice, with some more experienced clinicians less likely to adopt them than less experienced colleagues [28, 29, 34].

*“I know the NICE criteria, I know what I’m sort of thinking after examining the patient, I’m still doing it kind of in an old-fashioned way, still relying on what I think it is rather than the artificial intelligence side.”* [GP] [34]

Another potential barrier was practical and related to the software. Having the tools integrated into primary care practice software systems was deemed an important means of encouraging, facilitating and expediting use [26, 27, 29]. In addition, implementation was facilitated when the tool was time efficient, non-disruptive to the course of a consultation and made recommendations that the clinicians had confidence in [30, 32].

*“…it’s super easy to use it because every time we open a patient who is in that category the window pops up. You can just check the window, and it gives you all the information that you can use—which is really good.”* [GP] [32]

## Discussion

To our knowledge, this is the first systematic review to identify risk assessment tools used in primary care that support the detection of undiagnosed pancreatic cancer and to synthesise patient and primary care professionals’ perspectives. Overall, the perceived impacts on clinical decision-making and patient consultations were positive, although there were some important areas of concern and implementation barriers highlighted. Notwithstanding our comprehensive and extensive search strategies, only ten relevant studies were identified covering the use of five risk assessment tools, for the detection of multiple cancers including pancreatic, but none for pancreatic cancer only. This is somewhat surprising given that at least 52 pancreatic cancer diagnostic prediction models are reported to have been developed [12], but the findings of this review suggest that the overwhelming majority have not yet been incorporated within risk assessment tools used and investigated in primary care. This finding is supported by previous research in relation to risk prediction models generally, with Hendricksen et al. [9] highlighting a marked mismatch between the numbers of publications on model development when compared to publications on their implementation.

Taking the UK as an example, it may be that the market dominance of the Macmillan electronic clinical decision-support tools for cancer (which include QCancer and eRAT tools) means that many other models have not been applied in primary care. QCancer and eRATs were incorporated into the most widely-used GP software systems in 2013, through a collaboration between BMJ Informatica, Cancer Research UK and the Department of Health and Social Care [13]. However, there is an ongoing feasibility study applying the Enriching New-onset Diabetes for Pancreatic Cancer (ENDPAC) model [46] in primary care in the UK, with results expected in 2025 [47].

Our synthesis highlighted that these tools were very often valued, particularly by less experienced clinicians, echoing the findings of previous reviews [7, 48]. The tools were seen to support clinical decision-making in a variety of ways such as encouraging guideline-consistent referrals. However, the recent cluster-randomised trial of FHT identified in this review found that it did not significantly increase the proportion of patients receiving guideline-concordant care [31], although the authors note a possible ceiling effect for the intervention given the high follow-up rate in the control group.

The ways in which these tools enhance patient-clinician consultations also featured prominently, for example by personalising risk communication and facilitating shared decision-making. This is similar to Bradley et al.’s [7], Burns et al.’s [14] and He et al*.*’s [15] review findings. The integration of the tools within existing software was regarded as important, and not being integrated has been highlighted previously as a major barrier to their clinical use [49]. However, there was scepticism amongst some primary care professionals, particularly those with more experience, who raised concerns relating to how their use may increase the required length of consultations. This was also reported by Bradley et al. [7] and He et al*.* [15]. Whilst this is supported by the findings of a recent observational study using a subset of eRATs (not including pancreatic cancer), which found that individual consultations were significantly longer by almost four minutes, overall clinical session times did not increase significantly [50]. Concerns about the clinical validity of the tools were particularly prominent amongst experienced clinicians. This fits with the emphasis that previous research has placed on the role that their ‘gut instinct’ and experience plays in diagnosis and clinical decision-making [7, 14].

Participants’ experiences and perspectives differed across and within studies highlighting individual differences in the perceived value of these tools. For example, whilst some clinicians felt the process reassured their patients and expedited the consultation process, others raised concerns about increased consultation time and potentially causing unnecessary anxiety for their patients. There were similar divergences in opinions amongst patients with some saying they would prefer to be told about the use of the tool, even if it potentially increased their worry. Similarly, He et al*.*’s [15] review highlighted that patients could be both reassured and made more anxious by these tools. This underlines the need for clinicians to be aware of and sensitive to individual patient concerns and preferences with regard to the use of these tools. Whilst these tools were seen to remind clinicians of and to encourage adherence to guidelines, this was simultaneously suspected to both cause and reduce over-referrals, even amongst participants in the same study [26, 27]. However, Price et al. [33], focussing on QCancer and eRATs, found no difference in mean referral rates between practices with and without these cancer risk assessment tools, suggesting concerns about over-referrals may be unfounded. Further research into these tools’ impact on referrals is being undertaken as part of a randomised controlled trial to assess the clinical and cost effectiveness of the eRATs in general practice which will help in understanding this potential issue [51].

### Strengths and Limitations

One of the strengths of this review is that we have brought together the most recent, international, qualitative and quantitative literature relating specifically to the use of tools in primary care that can aid the pancreatic cancer diagnostic process. Using a comprehensive and systematic search process, we have synthesised previous research using a range of methods and highlighted their perceived value and the challenges to their use. This review therefore provides a consolidated resource for researchers and professionals interested in the diagnosis of pancreatic cancer in primary care and valuable information for those developing or refining such primary care risk assessment tools for this devastating disease. Furthermore, this review is a step towards understanding how GPs interact with risk assessment tools and to identify barriers to implementation and uptake, a need highlighted recently by Medina-Lara et al. [13]. Our review’s finding of how these tools can enhance patient consultations in a wide variety of ways, not only by helping with the understanding of risk, has not been emphasised in other relevant reviews.

However, the review has limitations. Firstly, only peer-reviewed literature was included, and all the studies came from developed nations (Australia, Israel and the UK); thus, the findings may not be generalisable to developing countries or to those with very different healthcare services and structures. In addition, there may be within-country differences in terms of, for example public and private primary care, and general practices with more resources and capacity for involvement in research and evaluation. This influences the likelihood of these practices undertaking and publishing investigations relevant to our review. Although most of the included qualitative studies scored well overall in quality assessment, concerns regarding sampling mean that their findings may not be transferrable to other populations. The inclusion of qualitative and quantitative research, whilst being more comprehensive compared to previous reviews, means the included studies also had a very wide range of aims, objectives and methodologies, resulting in challenges to synthesis. There may also be concerns about the transferability to real clinical consultations. Akanuwe et al*.* [26, 27] used vignettes to demonstrate how the tool could be used, and Chiang et al. [29] used actors to simulate consultations using vignettes, with a version of the QCancer Macmillan eCDS interface modified specifically for their study. In addition, as similarly reported by Burns et al. [14], there was much less emphasis on patient perspectives than those of clinicians. Even amongst those that did describe patient perspectives, the data reported were often less rich.

## Conclusions

Our review shows that these tools are perceived as potentially enhancing patient consultations and clinical decision-making. Consideration needs to be given to the design of the tools in relation to how this impacts their use in primary care. This is particularly important given the multiple demands on primary care professionals’ time and the potential wide-reaching benefits a well-designed tool could bring in this context. Despite an inclusive, exhaustive search of the literature, there is little evidence for the use of many of the developed pancreatic cancer diagnostic prediction models in primary care, suggesting there is scope for a dedicated pancreatic cancer risk assessment tool incorporating one of these previously developed models. Ideally, the tools need to be time efficient, compatible with the flow of the consultation, with clear evidence presented of validity and also integrated into patient record management systems. Future research should be undertaken to assess the clinical validity of these tools in order to encourage their uptake in clinical practice. Those developing such tools in the future can use the findings of this review to inform their tool development.

## Supplementary Information

Below is the link to the electronic supplementary material.

ESM 1 (DOCX 23.1 KB)

ESM 2 (PNG 367 KB)

ESM 2 (PNG 213 KB)

## Data Availability

No datasets were generated or analysed during the current study.
