# Supplementary material for: Pancreatic Cancer Risk Assessment Tools in Primary Care: A Mixed Methods Systematic Review
Source: J Gastrointest Cancer. 2025 Jun 5;56(1):128. doi: 10.1007/s12029-025-01229-5 (PMC12141374; doi:10.1007/s12029-025-01229-5)
Supplement: Supplementary file 1 — (DOCX 23.1 KB) [file 12029_2025_1229_MOESM1_ESM.docx]

# Supplementary Files

## Article title

Pancreatic cancer risk assessment tools in primary care: a mixed methods systematic review

## Journal name

Journal of Gastrointestinal Cancer

## Authors

Hugh Claridge, School of Health Sciences, Faculty of Health and Medical Sciences, University of Surrey, Guildford, UK. Correspondence to: [hc01558@surrey.ac.uk](mailto:hc01558@surrey.ac.uk)

Elizabeth A. Cooke, National Physical Laboratory, Teddington, UK.

Spencer A. Thomas, National Physical Laboratory, Teddington, UK.

Nan Greenwood, Kingston University, Kingston, UK.

Agnieszka Lemanska, School of Health Sciences, Faculty of Health and Medical Sciences, University of Surrey, Guildford, UK.

## File 1: Setting Population Intervention Comparison Evaluation (SPICE) criteria [19]

| **Box 1: SPICE criteria** | |
| --- | --- |
| **S**etting | Primary care only, or with additional involvement of other settings such as for patient investigations. |
| **P**opulation or perspective | Primary care professionals and patients. |
| **I**ntervention | Tools with risk assessment functions that aid primary care clinicians in diagnosing pancreatic cancer. |
| **C**omparison | No comparison. |
| **E**valuation | Qualitative, quantitative or mixed methods investigations where these tools have been used. |

## File 2: Literature database search strings

**Web of Science search string**

1: TS=("risk assess*" OR "risk score*" OR "risk tool*" OR "risk stratif*" OR algorithm* OR "decision model*" OR "diagnosis model*" OR "diagnostic model*" OR "support model*" OR "screening model*" OR "decision tool*" OR "diagnosis tool*" OR "diagnostic tool*" OR "support tool*" OR "screening tool*" OR "computer* tool*" OR "electronic tool*" OR "decision aid*" OR "diagnosis aid*" OR "diagnostic aid*" OR "support aid*" OR "screening aid*" OR "computer* aid*" OR "electronic aid*"OR "decision system*" OR "diagnosis system*" OR "diagnostic system*" OR "screening system*" OR "decision support system*" OR "diagnosis support system*" OR "diagnostic support system*" OR "screening support system*" OR "decision prompt*" OR "diagnosis prompt*" OR "diagnostic prompt*" OR "support prompt*" OR "screening prompt*" OR "computer* prompt*" OR "electronic prompt*" OR "decision technolog*" OR "diagnosis technolog*" OR "diagnostic technolog*" OR "support technolog*" OR "screening technolog*" OR "information technolog*" OR "assistive technology*" OR "assisted diagnos*")

2: TS=(neoplas* OR carcinoma* OR cancer* OR tumo$r OR malignan* OR adenocarcinoma* OR adenoma*)

3: TS=("primary care" OR "primary health*" OR "primary health care" OR "primary healthcare" OR "general practic*" OR GP OR "family doctor*" OR "family medic*" OR "family practi*" OR "community pharmac*" OR "community nurs*" OR "ambulatory care" OR "doctor* surger*" OR "doctor* office*" OR "local surger*")

4: TS=(trial* OR pilot* OR feasib* OR implement* OR deploy* OR adopt* OR use* OR utili* OR evaluati* OR interven* OR barrier* OR facilitat* OR perspective* OR perceive* OR perception* OR experience* OR attitude* OR acceptab*)

5: (#1 AND #2 AND #3 AND #4)

**Ovid Embase search string**

1: ("risk assess*" or "risk score*" or "risk tool*" or "risk stratif*" or algorithm* or "decision model*" or "diagnosis model*" or "diagnostic model*" or "support model*" or "screening model*" or "decision tool*" or "diagnosis tool*" or "diagnostic tool*" or "support tool*" or "screening tool*" or "computer* tool" or "electronic tool*" or "decision aid*" or "diagnosis aid*" or "diagnostic aid*" or "support aid*" or "screening aid*" or "computer* aid*" or "electronic aid*OR decision system*" or "diagnosis system*" or "diagnostic system*" or "screening system*" or "decision support system*" or "diagnosis support system*" or "diagnostic support system*" or "screening support system*" or "decision prompt*" or "diagnosis prompt*" or "diagnostic prompt*" or "support prompt*" or "screening prompt*" or "computer* prompt*" or "electronic prompt*" or "decision technolog*" or "diagnosis technolog*" or "diagnostic technolog*" or "support technolog*" or "screening technolog*" or "information technolog*" or "assistive technology*" or "assisted diagnos*").mp. [mp=title, abstract, heading word, drug trade name, original title, device manufacturer, drug manufacturer, device trade name, keyword heading word, floating subheading word, candidate term word]

2: (neoplas* or carcinoma* or cancer* or tumo$r or malignan* or adenocarcinoma* or adenoma*).mp. [mp=title, abstract, heading word, drug trade name, original title, device manufacturer, drug manufacturer, device trade name, keyword heading word, floating subheading word, candidate term word]

3: ("primary care" or "primary health*" or "primary health care" or "primary healthcare" or "general practic*" or GP or "family doctor*" or "family medic*" or "family practi*" or "community pharmac*" or "community nurs*" or "ambulatory care" or "doctor* surger*" or "doctor* office*" or "local surger*").mp. [mp=title, abstract, heading word, drug trade name, original title, device manufacturer, drug manufacturer, device trade name, keyword heading word, floating subheading word, candidate term word]

4: (trial* or pilot* or feasib* or implement* or deploy* or adopt* or use* or utili* or evaluati* or interven* or barrier* or facilitat* or perspective* or perceive* or perception* or experience* or attitude* or acceptab*).mp. [mp=title, abstract, heading word, drug trade name, original title, device manufacturer, drug manufacturer, device trade name, keyword heading word, floating subheading word, candidate term word]

5: 1 and 2 and 3 and 4

**Cochrane Library search string**

1: ((risk NEXT assess*) OR (risk NEXT score*) OR (risk NEXT tool*) OR (risk NEXT stratif*) OR algorithm* OR (decision NEXT model*) OR (diagnosis NEXT model*) OR (diagnostic NEXT model*) OR (support NEXT model*) OR (screening NEXT model*) OR (decision NEXT tool*) OR (diagnosis NEXT tool*) OR (diagnostic NEXT tool*) OR (support NEXT tool*) OR (screening NEXT tool*) OR (computer* NEXT tool*) OR (electronic NEXT tool*) OR (decision NEXT aid*) OR (diagnosis NEXT aid*) OR (diagnostic NEXT aid*) OR (support NEXT aid*) OR (screening NEXT aid*) OR (computer* NEXT aid*) OR (electronic NEXT aid*) OR (decision NEXT system*) OR (diagnosis NEXT system*) OR (diagnostic NEXT system*) OR (screening NEXT system*) OR (decision NEXT support NEXT system*) OR (diagnosis NEXT support NEXT system*) OR (diagnostic NEXT support NEXT system*) OR (screening NEXT support NEXT system*) OR (decision NEXT prompt*) OR (diagnosis NEXT prompt*) OR (diagnostic NEXT prompt*) OR (support NEXT prompt*) OR (screening NEXT prompt*) OR (computer* NEXT prompt*) OR (electronic NEXT prompt*) OR (decision NEXT technolog*) OR (diagnosis NEXT technolog*) OR (diagnostic NEXT technolog*) OR (support NEXT technolog*) OR (screening NEXT technolog*) OR (information NEXT technolog*) OR (assistive NEXT technology*) OR (assisted NEXT diagnos*)):ti,ab,kw (Word variations have been searched)

2: neoplas* OR carcinoma* OR cancer* OR tumo$r OR malignan* OR adenocarcinoma* OR adenoma*

3: (primary NEXT care) OR (primary NEXT health*) OR (primary NEXT health NEXT care) OR (primary NEXT healthcare) OR (general NEXT practic*) OR GP OR (family NEXT doctor*) OR (family NEXT medic*) OR (family NEXT practi*) OR (community NEXT pharmac*) OR (community NEXT nurs*) OR (ambulatory NEXT care) OR (doctor* NEXT surger*) OR (doctor* NEXT office*) OR (local NEXT surger*)

4: trial* OR pilot* OR feasib* OR implement* OR deploy* OR adopt* OR use* OR utili* OR evaluati* OR interven* OR barrier* OR facilitat* OR perspective* OR perceive* OR perception* OR experience* OR attitude* OR acceptab*

#5: #1 AND #2 AND #3 AND #4

## Supplementary File 3: QualSyst Checklists [27]

**Checklist for assessing the quality of qualitative studies**

| **Criteria** |  | **YES**  **(2)** | **PARTIAL**  **(1)** | **NO**  **(0)** |
| --- | --- | --- | --- | --- |
| 1 | Question / objective sufficiently described? |  |  |  |
| 2 | Study design evident and appropriate? |  |  |  |
| 3 | Context for the study clear? |  |  |  |
| 4 | Connection to a theoretical framework / wider body of knowledge? |  |  |  |
| 5 | Sampling strategy described, relevant and justified? |  |  |  |
| 6 | Data collection methods clearly described and systematic? |  |  |  |
| 7 | Data analysis clearly described and systematic? |  |  |  |
| 8 | Use of verification procedure(s) to establish credibility? |  |  |  |
| 9 | Conclusions supported by the results? |  |  |  |
| 10 | Reflexivity of the account? |  |  |  |

**Checklist for assessing the quality of quantitative studies**

| Criteria |  | YES  (2) | PARTIAL  (1) | NO  (0) | N/A |
| --- | --- | --- | --- | --- | --- |
| 1 | Question / objective sufficiently described? |  |  |  |  |
| 2 | Study design evident and appropriate? |  |  |  |  |
| 3 | Method of subject/comparison group selection *or* source of information/input variables described and appropriate? |  |  |  |  |
| 4 | Subject (and comparison group, if applicable) characteristics sufficiently described? |  |  |  |  |
| 5 | If interventional and random allocation was possible, was it described? |  |  |  |  |
| 6 | If interventional and blinding of investigators was possible, was it reported? |  |  |  |  |
| 7 | If interventional and blinding of subjects was possible, was it reported? |  |  |  |  |
| 8 | Outcome and (if applicable) exposure measure(s) well defined and robust to measurement / misclassification bias?  Means of assessment reported? |  |  |  |  |
| 9 | Sample size appropriate? |  |  |  |  |
| 10 | Analytic methods described/justified and appropriate? |  |  |  |  |
| 11 | Some estimate of variance is reported for the main results? |  |  |  |  |
| 12 | Controlled for confounding? |  |  |  |  |
| 13 | Results reported in sufficient detail? |  |  |  |  |
| 14 | Conclusions supported by the results? |  |  |  |  |
