# Supplementary figures and images for: Pancreatic Cancer Risk Assessment Tools in Primary Care: A Mixed Methods Systematic Review
Source: J Gastrointest Cancer. 2025 Jun 5;56(1):128. doi: 10.1007/s12029-025-01229-5 (PMC12141374; doi:10.1007/s12029-025-01229-5)

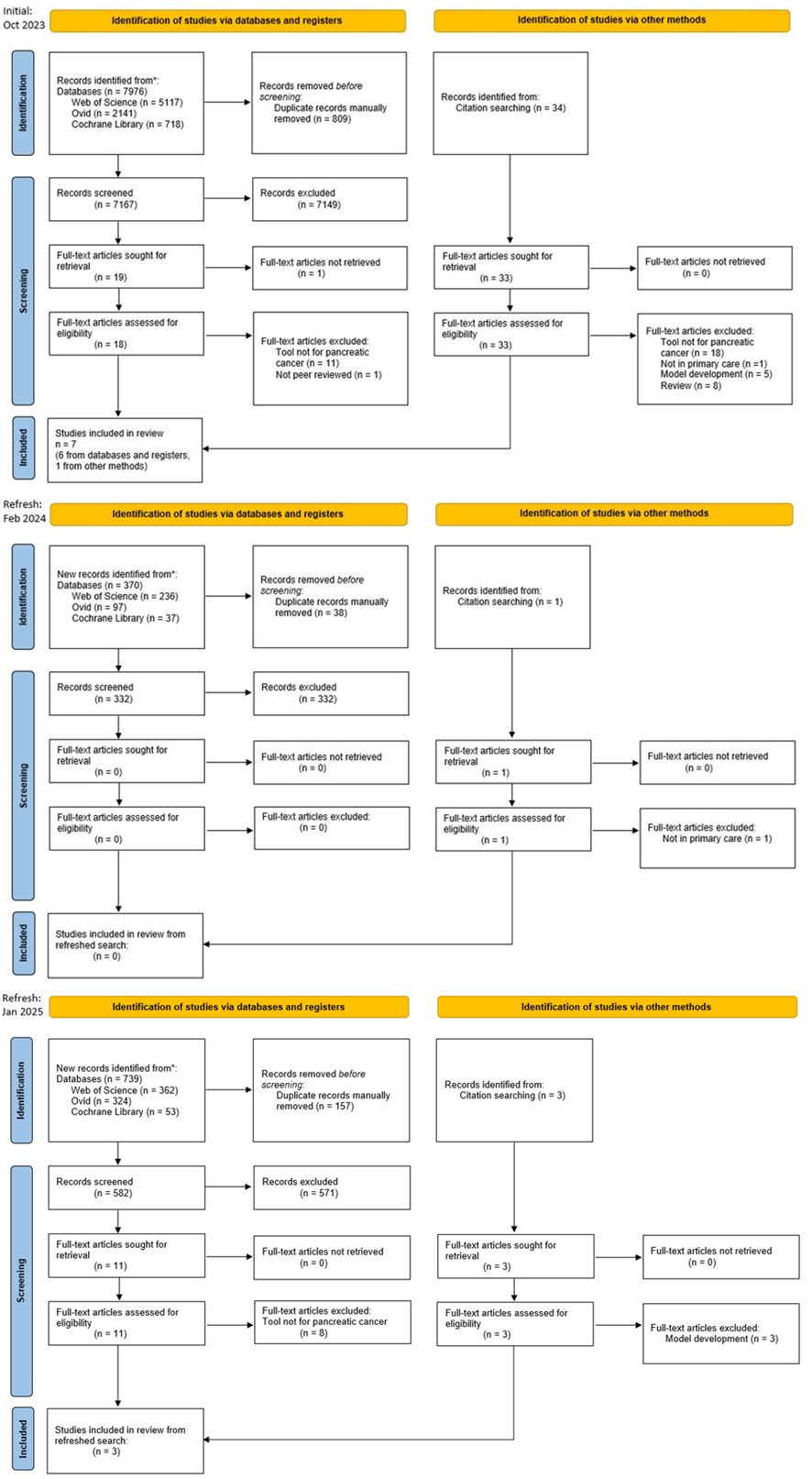

Supplement: Supplementary file 2 — (PNG 367 KB) [file 12029_2025_1229_MOESM2_ESM.png]

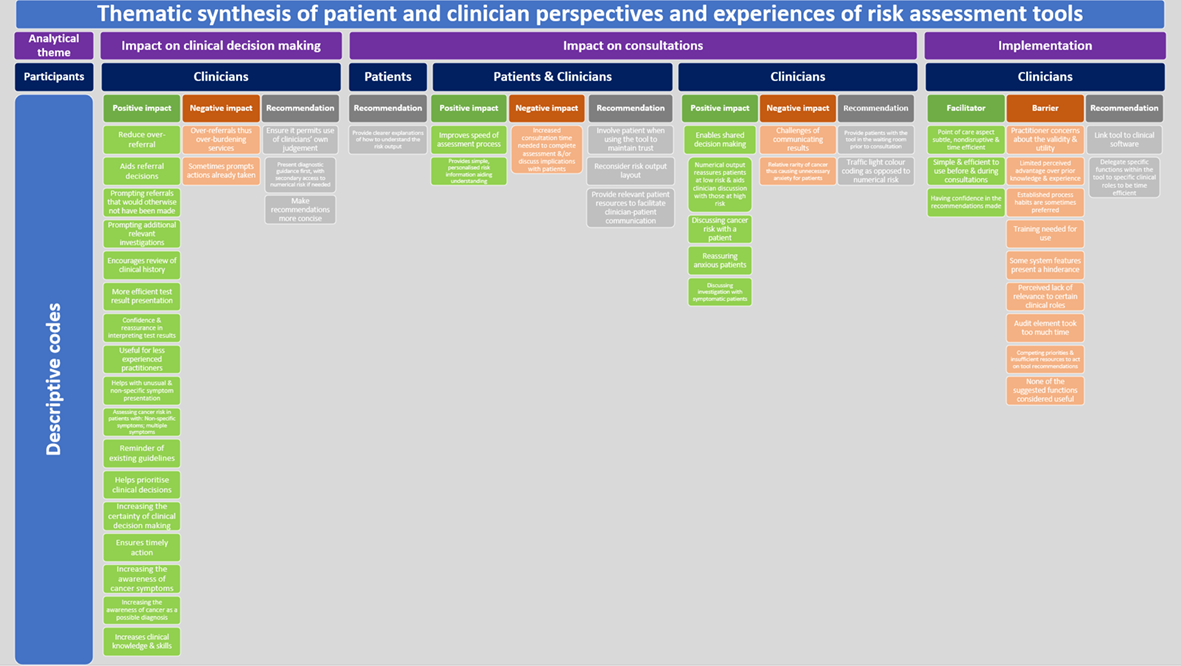

Supplement: Supplementary file 3 — (PNG 213 KB) [file 12029_2025_1229_MOESM3_ESM.png]
